# Combining cerebrospinal fluid and PI‐2620 tau‐PET for biomarker‐based stratification of Alzheimer's disease and 4R‐tauopathies

**DOI:** 10.1002/alz.14185

**Published:** 2024-09-12

**Authors:** Roxane Dilcher, Stephan Wall, Mattes Groß, Sabrina Katzdobler, Olivia Wagemann, Carla Palleis, Endy Weidinger, Urban Fietzek, Alexander Bernhardt, Carolin Kurz, Christian Ferschmann, Maximilian Scheifele, Mirlind Zaganjori, Johannes Gnörich, Katharina Bürger, Daniel Janowitz, Boris‐Stephan Rauchmann, Sophia Stöcklein, Peter Bartenstein, Victor Villemagne, John Seibyl, Osama Sabri, Henryk Barthel, Robert Perneczky, Florian Schöberl, Andreas Zwergal, Günter U. Höglinger, Johannes Levin, Nicolai Franzmeier, Matthias Brendel

**Affiliations:** ^1^ Neuroscience Monash University Melbourne Australia; ^2^ Department of Nuclear Medicine University Hospital of Munich LMU Munich München Germany; ^3^ Institute for Stroke and Dementia Research University Hospital of Munich, LMU Munich München Germany; ^4^ (SyNergy), Munich Cluster for Systems Neurology München Germany; ^5^ Department of Neurology University Hospital of Munich, LMU Munich München Germany; ^6^ (DZNE), German Center for Neurodegenerative Diseases München Germany; ^7^ Department of Psychiatry and Psychotherapy University Hospital of Munich, LMU Munich München Germany; ^8^ Institute for Neuroradiology University Hospital of Munich, LMU Munich München Germany; ^9^ Department of Radiology University Hospital of Munich, LMU Munich München Germany; ^10^ Department of Psychiatry University of Pittsburgh Pittsburgh Pennsylvania USA; ^11^ Department of Molecular Imaging & Therapy Austin Health Heidelberg Australia; ^12^ Institute for Neurodegenerative Disorders New Haven Connecticut USA; ^13^ Department of Nuclear Medicine University Hospital Leipzig Leipzig Germany; ^14^ German Center for Vertigo and Balance Disorders (DSGZ) University Hospital of Munich, LMU Munich München Germany; ^15^ Department of Psychiatry and Neurochemistry University of Gothenburg The Sahlgrenska Academy Institute of Neuroscience and Physiology Mölndal and Gothenburg Sweden

**Keywords:** ^18^F‐PI‐2620, 4‐repeat tauopathies, Alzheimer's disease, biomarkers, cerebrospinal fluid, perfusion, PET, tau

## Abstract

**INTRODUCTION:**

Recent advances in biomarker research have improved the diagnosis and monitoring of Alzheimer's disease (AD), but in vivo biomarker‐based workflows to assess 4R‐tauopathy (4RT) patients are currently missing. We suggest a novel biomarker‐based algorithm to characterize AD and 4RTs.

**METHODS:**

We cross‐sectionally assessed combinations of cerebrospinal fluid measures (CSF p‐tau_181_ and t‐tau) and ^18^F‐PI‐2620 tau‐positron emission tomography (PET) in patients with AD (*n* = 64), clinically suspected 4RTs (progressive supranuclear palsy or corticobasal syndrome, *n* = 82) and healthy controls (*n* = 19).

**RESULTS:**

Elevated CSF p‐tau_181_ and cortical ^18^F‐PI‐2620 binding was characteristic for AD while normal CSF p‐tau_181_ with elevated subcortical ^18^F‐PI‐2620 binding was characteristic for 4RTs. ^18^F‐PI‐2620‐assessed posterior cortical hypoperfusion could be used as an additional neuronal injury biomarker in AD.

**DISCUSSION:**

The specific combination of CSF markers and ^18^F‐PI‐2620 tau‐PET in disease‐specific regions facilitates the biomarker‐guided stratification of AD and 4RTs. This has implications for biomarker‐aided diagnostic workflows and the advancement in clinical trials.

**Highlights:**

- Novel biomarker‐based algorithm for differentiating AD and 4R‐tauopathies.
- A combination of CSF p‐tau_181_ and ^18^F‐PI‐2620 discriminates AD versus 4R tauopathies.
- Hypoperfusion serves as an additional neuronal injury biomarker in AD.

## 1 BACKGROUND

Tauopathies are a group of neurodegenerative diseases characterized by the abnormal aggregation of the microtubule‐associated protein tau in neurons and glial cells. In tauopathies, the aggregation of tau pathology has been suggested to follow disease‐specific spatio‐temporal spreading patterns^1^, ^2^ and to causally drive neurodegeneration and therefore symptom progression.^3^, ^4^ Tauopathies are typically classified into primary and secondary tauopathies based on the molecular composition of tau aggregates and the co‐occurrence of other protein deposits, such as beta amyloid plaque pathology in Alzheimer's disease (AD). The primary tauopathies progressive supranuclear palsy (PSP) or corticobasal degeneration (CBD) are characterized by an abnormal aggregation of tau isoforms with four‐repeat binding domains within neurons and glial cells typically in brainstem regions and subcortical nuclei, which manifest as atypical parkinsonian syndromes with oculo‐motor symptoms, gait disturbance, and falls in PSP, as well as asymmetric hypokinetic rigid syndrome, limb dyspraxia, and alien limb phenomenon in CBD.^5^ In contrast, AD is a secondary beta‐amyloid associated tauopathy characterized by the intraneuronal aggregation of 3/4R‐tau in the cortex rather than subcortex, causing cognitive rather than motor symptoms.^6^ In recent years, there have been remarkable advances in biomarker‐based diagnosis and monitoring of AD as a secondary 3/4R‐tauopathy (4RT), with specific biomarkers that can detect beta amyloid and tau aggregates in vivo via positron emission tomography (PET) imaging or determine pathophysiological amyloid and tau species in cerebrospinal fluid (CSF) or blood plasma. These advances in biomarker development have helped establish biomarker‐based staging systems (i.e., ATN,^7^ Amyloid/Tau/Neurodegeneration), biomarker‐based diagnostic workflows, and the implementation of biomarker endpoints in clinical trials assessing disease‐modifying treatments.

In contrast, the primary 4RTs PSP and CBD are still purely diagnosed using clinical criteria (e.g., PSP–Richardson Syndrome, corticobasal syndrome [CBS]) with supportive MRI‐based neurodegeneration patterns.^8^ Yet, definite diagnosis can only be made post‐mortem, since no specific imaging or fluid biomarker for 4RTs is widely established in clinical practice. The lack of specific 4RT biomarkers clearly limits in vivo disease diagnosis, a mechanistic understanding of disease progression, as well as the development of treatments and monitoring of pathophysiological disease progression. The recently introduced second generation tau‐PET tracer ^18^F‐PI‐2620 has been shown to have affinity to 4R‐tau deposits as well as 3/4R‐tau in autoradiography and immunohistochemistry, together with an improved off‐target binding profile,^9^ suggesting suitability as a tau biomarker across primary and secondary tauopathies. Supporting this, we and others have shown that ^18^F‐PI‐2620 tau‐PET shows elevated in vivo binding in 4R‐tau target regions in the basal ganglia in patients with PSP and CBS.^10^, ^11^ Moreover, it has the potential to serve as a surrogate biomarker for neuronal injury in tauopathies by showing reduced early‐phase ^18^F‐PI‐2620 perfusion.^12^, ^13^ Here, we suggest a novel diagnostic algorithm, which is based on biological markers, including CSF, tau‐PET, and perfusion imaging to characterize and differentiate primary and secondary tauopathies, and which would have implications for future clinical diagnosis and for the advancement in clinical trials.

Specifically, we combined ^18^F‐PI‐2620 PET imaging and fluid (CSF p‐tau_181_/t‐tau) markers in 64 patients with AD (i.e., amyloid positive) and 82 patients with clinically suspected 4RTs to establish biomarker‐based diagnostic workflows for characterizing patients with AD versus 4RT. To this end, we first aimed to identify disease‐specific pattern differences in ^18^F‐PI‐2620 tau binding and ^18^F‐PI‐2620 hypoperfusion for AD versus 4RT. Second, we assessed whether CSF markers of tau pathophysiology or neuronal injury were elevated in AD but not in 4RT, given that p‐tau_181_ and t‐tau are considered to be more elevated in AD compared to 4RT. Last, we determined the unique and combined performance of tau‐PET and fluid markers to discriminate AD versus 4RT and controls.

**RESEARCH IN CONTEXT**

1. **Systematic review**: We reviewed the most recent and most relevant literature using traditional sources (e.g., PubMed) to evaluate existing biomarkers in Alzheimer's disease (AD) and 4R‐tauopathies (4RTs). There have been advances in the implementation of biomarkers in diagnostic and in clinical trials in AD, but there is a lack of in vivo biomarkers for primary 4RTs.
2. **Interpretation**: In this hypothesis‐driven study, we suggest a biomarker‐based diagnostic algorithm to differentiate between AD and 4RTs, by using a positron emission tomography (PET) tracer with 4R tau affinity in combination with cerebrospinal fluid. A new biomarker‐based algorithm can add to existing clinical workflows for the assessment of 4RTs.
3. **Future directions**: We recommend implementing the current findings into longitudinal studies to test the biomarker's performance to track disease progression. A robust, biomarker‐based algorithm can be used as endpoint in future clinical trials for the assessment of disease‐modifying treatments in primary 4RTs.

## 2 METHODS

### 2.1 Patient enrolment and sample characteristics

This cross‐sectional study included 64 patients on the AD spectrum (positive on amyloid biomarkers), 82 with a clinically suspected 4RT (PSP or CBS, negative on amyloid biomarkers), and 19 age‐ and sex‐matched healthy controls. All patients were referred by dementia or movement disorder experts of a tertiary center to ^18^F‐PI‐2620 tau‐PET imaging and recruited and scanned at the department of nuclear medicine at the Ludwig Maximilian University in Munich. Data were collected from October 2018 to May 2024. The most likely clinical diagnosis was given before performing ^18^F‐PI‐2620 tau‐PET imaging. Patients with typical AD were required to meet criteria for typical AD with mild cognitive impairment (MCI) or dementia according to the diagnostic criteria of the National Institute on Aging and Alzheimer's Association and only included as AD if CSF or PET biomarkers of beta amyloid pathology were positive. Diagnosis of 4RTs was made according to the revised Armstrong Criteria of probable CBS or the Movement Disorders Society criteria of possible/probable PSP or possible PSP with predominant CBS.^8^, ^14^ Aβ concentration and Aβ ratio in CSF and/or ^18^F‐flutemetamol PET (at least one was available) served for assessment of the Aβ status. The 4RT classification required an Aβ‐negative profile. If those patients were tested positive for Aβ in CSF or PET, they were classified as having AD (i.e., AD‐CBS), comprising a subset of 15 patients. All patients (or their legal representatives) provided informed written consent prior to PET scanning. The study was conducted in accordance with the principles of the Declaration of Helsinki. Approval for scientific data analysis was obtained from the local ethics committee (application numbers 17‐569, 19‐022).

### 2.2 CSF analyses

A subgroup of participants (AD: *n *= 54, 4RT: *n* = 53; controls: *n* = 9) underwent lumbar puncture at their visit at LMU University Hospital, Munich, and their CSF levels were analyzed with enzyme‐linked immunosorbent assay (ELISA) Innotest Kit (Fujirebio Europe N.V., Belgium) at the MVZ laboratory PD Dr. Volkmann & Kollegen GbR in Karlsruhe, Germany. The CSF biomarkers included Aβ_42_, Aβ_40_, Aβ ratio, t‐tau, tau/Aβ ratio, and p‐tau_181_. The respective normal cutoff values were for Aβ_42_ > 375 pg/mL, Aβ ratio > 5.5%, t‐tau < 445 pg/mL, t‐tau/Aβ ratio < 1000, and for p‐tau_181_ < 61 pg/mL, according to standardized laboratory diagnostics at the partnering laboratory.

### 2.3 PET imaging acquisition and preprocessing

^18^F‐PI‐2620 PET was performed on SIEMENS cameras (i.e., Biograph 64 or mCT PET/CT, SIEMENS, Erlangen, Germany) using a full dynamic 0–60‐min recording protocol. Computed tomography was used for attenuation correction. PET data were reconstructed in a series of 35 frames. Details of image acquisition procedures have been described previously.^10^ All imaging data were processed using a fully automated in‐house processing pipeline. Each full dynamic dataset (0–60 min.) was motion‐corrected using rigid alignment. To determine the perfusion‐PET signal, we extracted a single static frame of early‐phase perfusion (frame 0.5–2.5 min. after injection) which was subsequently non‐linearly normalized to a tracer‐specific template in the Montreal Neurological Institute (MNI) space for spatial normalization. Normalization included non‐linear warping, 8 mm input smoothing, equal modality, 16 iterations, frequency cutoff 3, regularization 1.0, and no thresholding. The transformation was applied to the full dynamic ^18^F‐PI‐2620 PET datasets to minimize interpolation. Perfusion‐PET images were standardized uptake value ratio (SUVr) normalized to the mean intensity of a pons reference region determined in MNI space. For assessing tau‐specific PET binding, distribution volume ratios (DVR) were assessed on motion‐corrected dynamic ^18^F‐PI‐2620 data using the simplified reference tissue model 2 as implemented in the Qmodeling package^15^ using the inferior cerebellar grey matter as reference region. Resulting DVR images were also non‐linearly normalized to a tracer‐specific template in MNI space using the settings described above. For region of interest (ROI) analyses, DVRs, and perfusion SUVrs were extracted in MNI space for the Brainnetome atlas.^16^ To determine perfusion‐ or tau‐PET abnormality, Z‐scores were obtained for DVR and SUVr values, using the *n* = 19 healthy control subjects, with the following formula:

$$z-\mathrm{score}=\frac{\mathrm{patient}\;\mathrm{DVR}\;\left(\mathrm{or}\;\mathrm{SUVr}\right)-\mathrm{mean}\;\mathrm{of}\;\mathrm{controls}}{\mathrm{SD}\;\mathrm{of}\;\mathrm{controls}}$$

For comparative analysis of perfusion‐PET, voxel‐wise R1 images were additionally assessed using the simplified reference tissue model 2 to measure neuronal injury. For comparative analysis of tau binding, voxel‐wise late‐phase SUVr images (frame 20–40 min.) were additionally analyzed to evaluate tau‐specific PET binding. The results of these assessments are presented in Figure S1. Exemplary time‐activity‐curves of ^18^F‐PI‐2620 are presented in Figure S2.

### 2.4 Statistics

Statistical analyses on ROI‐level data were carried out with R (R Core Team, 2022), voxel‐wise analyses were performed with SPM12^15^ and VoxelStats^17^ in Matlab. Cortical and subcortical ROIs were derived from the Brainnetome atlas. All voxel‐wise and ROI‐based analyses used age and sex as covariates and were tested at the *p *< 0.001 significance level.

To assess different tau binding or perfusion characteristics between AD or 4RT and controls, we performed a two‐sample *t*‐test for voxel‐wise analyses and analyses of covariance (ANCOVAs) for ROI‐based DVR/SUVr_0.5–2.5_ differences. To calculate group differences in p‐tau_181_ and t‐tau, we performed ANCOVAs. Group differences in clinical scores were assessed by ANOVA and chi‐squared‐test. Next, only patients with available CSF samples were analyzed to assess associations between tau markers p‐tau_181_ and tau‐PET binding (i.e., DVR) or between neuronal injury markers t‐tau and perfusion (SUVr_0.5–2.5_) by using voxel‐wise and ROI‐based multiple regression analyses in each group. We also tested for interactions, to see if the relationship between p‐tau_181_ and DVR, or between t‐tau and SUVr_0.5–2.5_ differed between AD and 4RT, using a voxel‐wise flexible factorial design and an ROI‐based multiple regression analysis with interaction. To further look at association patterns, we calculated the percentage of cases belonging to specific quadrants of a scatterplot. For the tau binding data, an abnormality cutoff of +1 SD above the mean of controls was set for the z‐transformed DVRs, and a cutoff of 61 pg/mL for the p‐tau_181_ levels. For the perfusion data, an abnormality cutoff of −1 was set for the SUVr z‐scores and a cutoff of 445 pg/mL for t‐tau. Density plots revealed that cutoffs of +1 (DVR z‐score) and −1 (SUVr z‐score) effectively separated diagnostic groups from controls, minimizing false negatives and increasing the sensitivity of the analysis (Figure S3).

Post‐hoc analyses were performed using Tukey adjustment. Cohen's d for effect sizes and confidence intervals are reported. False‐discovery rate (FDR) corrections for multiple comparisons between groups were performed for the voxel‐wise and ROI‐based analyses.

To address the discriminatory performance on regional tau binding or perfusion between AD versus 4RT/controls, or between 4RT versus AD/controls, we performed voxel‐wise receiver operating characteristic (ROC) analyses in VoxelStats to test the discriminatory power at each voxel. Areas that showed the largest tau binding or perfusion difference between AD versus controls, and between 4RT versus controls, were used for subsequent ROI‐based ROC analyses. The area under the curve (AUC) values were compared between biomarker combinations, by adding p‐tau_181_ with regional tau binding (tau biomarker measure), and t‐tau with regional perfusion (neuronal injury measure). Finally, classification decision tree analysis was utilized to investigate the additional value of neuronal injury biomarkers when combined with tau biomarkers.

## 3 RESULTS

The overall sample included 165 participants, that is, 64 patients with AD (age: 73.1 ± 8.3 years, 34 females, 30 males), 82 patients with 4RT (age: 71.5 ± 7.0 years, 33 females, 49 males) of which 58 were diagnosed with PSP and 24 with CBS, and 19 healthy controls (age: 67.9 ± 11.1, 8 females, 11 males). A subset of 116 participants had available CSF measures (AD: *n *= 54, 4RT: *n *= 53; controls: *n *= 9). Details are provided in Table 1.

**TABLE 1:** Demographics and clinical assessment.

Parameter	AD (*n* = 64)	PSP (*n *= 58)	CBS (*n *= 24)	Controls (*n *= 19)	*p*‐value
Age, mean (SD)	73.1 (8.3)	71.1 (7.1)	72.2 (6.7)	67.9 (11.1)	0.075
Female, *n* (%)	34 (53)	25 (43)	8 (33)	8 (42)	0.368
Male, *n* (%)	30 (47)	33 (57)	16 (67)	11 (58)	
Disease duration, months (SD)	29.2 (16.5) (*n* = 24)	36.1 (29.1) (*n* = 47)	34.1 (22.2) (*n* = 19)	/	0.449
MoCA score, mean (SD)	17.6 (6.4) (*n* = 25)	21.9 (4.8) (*n* = 47)	22.6 (4.4) (*n* = 21)	27.8 (2.1) (*n* = 4)	**0.000** ^***^
PSPRS, mean (SD)	23.4 (6.7) (*n* = 9)	31.6 (9.4) (*n* = 47)	23.6 (13.0) (*n* = 16)	/	**0.008** ^**^
UPDRS, mean (SD)	32.6 (11.1) (*n* = 8)	34.8 (13.3) (*n* = 38)	35.3 (15.9) (*n* = 16)	/	0.775
SEADL, mean (SD)	66.7 (13.2) (*n* = 9)	60.4 (19.0) (*n* = 46)	66.3 (16.3) (*n* = 16)	/	0.346
CSF, *n* (%)	54 (84)	32 (55)	21 (88)	9 (47)	
p‐tau, mean pg/mL (SD)	82.7 (37)	50.9 (32.9)	50.7 (20.1)	52.0 (19.0)	**0.000** ^***^
t‐tau, mean pg/mL (SD)	479 (259)	251 (211)	299 (179)	242 (108)	**0.000** ^***^

*Note*: Bold values indicate statistically significant results at the *p* < 0.05 level.

Abbreviations: AD, Alzheimer's disease; CBS, corticobasal syndrome, CSF, cerebrospinal fluid; MoCA, Montreal Cognitive Assessment; PSP, progressive supranuclear palsy; PSPRS, Progressive Supranuclear Palsy Rating Scale; SD, standard deviation; SEADL, Schwab and England Activities of Daily Living; UPDRS, Unified Parkinson's Disease Rating Scale.

* *p* < 0.05.

** *p* < 0.01.

*** *p* < 0.001.

### 3.1 Detection of cortical tau binding and perfusion in AD and subcortical tau binding in 4RT

**FIGURE 1:**
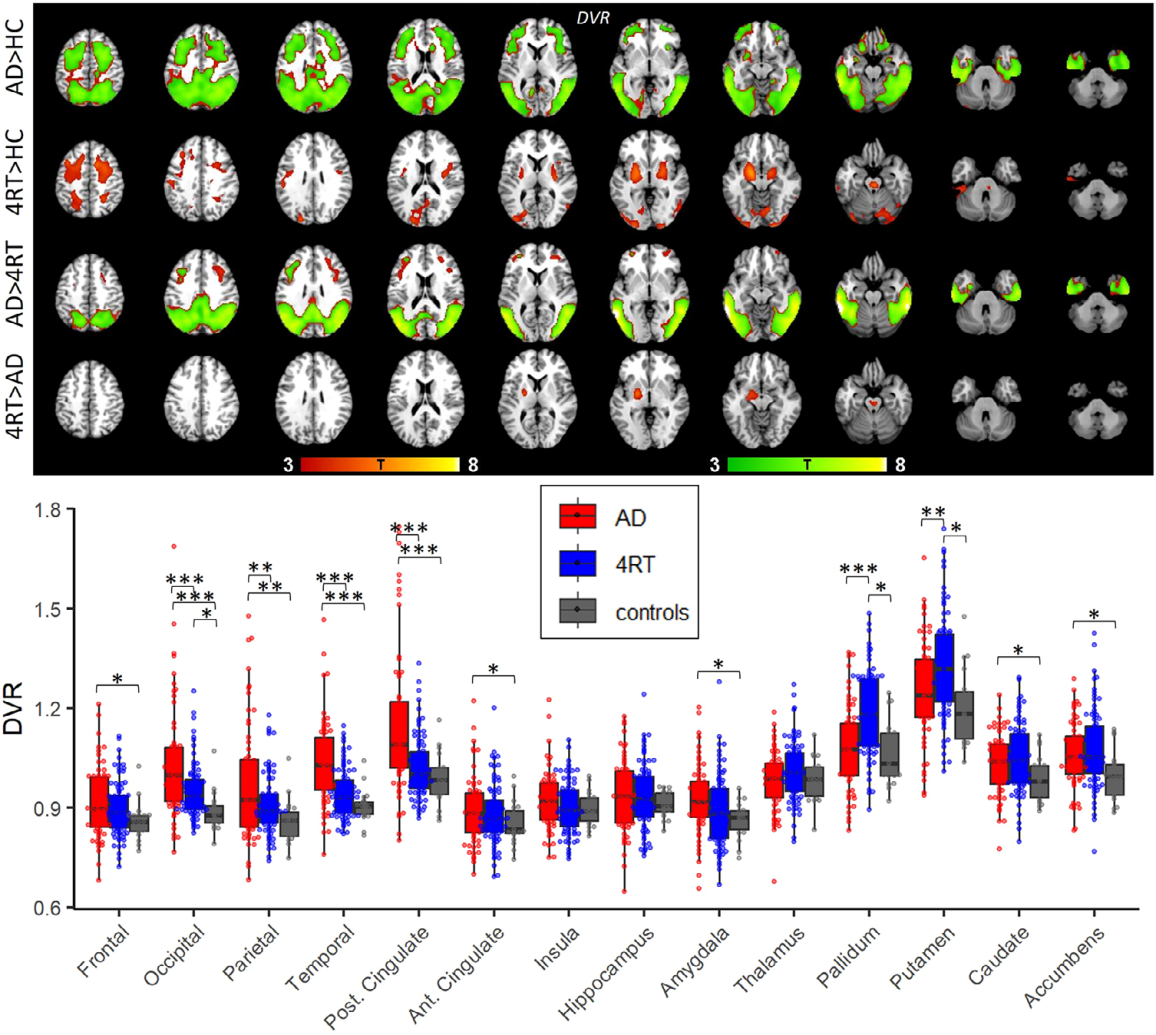
Group differences of tau binding. The top images show voxel‐wise group comparisons of tau binding. Green areas indicate FDR‐corrected voxels. Voxel‐wise T‐statistics are shown at *p *< 0.001, *k *> 500, controlled for age and sex, and overlaid on a standard template T1 MRI image. The bottom image shows DVR group comparisons in single regions of interests. DVR, distribution volume ratios; FDR, false discovery rate; MRI, magnetic resonance imaging.

First, tau‐PET binding characteristics based on DVR were compared between groups. Voxel‐wise *t*‐tests revealed significantly higher tau binding in AD compared to controls in frontal and posterior cortical areas (occipital, parietal, temporal) (*p *< 0.001, FDR corrected, *k *> 500), and significantly more tau binding in posterior cortical regions compared to 4RT (*p *< 0.001, FDR corrected, *k *> 500). The 4RT group showed significantly higher tau binding in the pallidum and putamen compared to controls (*p *< 0.001, uncorrected, *k *> 500) and in the left pallidum and left putamen when compared to AD (*p *< 0.001, uncorrected, *k *> 500) (Figure 1).

ROI analyses revealed significantly higher tau binding in AD compared to 4RT in posterior cortical regions (occipital, parietal, temporal, posterior cingulate) and compared to controls in posterior cortical regions, frontal, anterior cingulate, amygdala, caudate, and accumbens. The 4RT group showed significantly higher tau binding compared to AD and controls in the pallidum and putamen, and compared to controls additionally in the occipital lobe (Table 2, Figure 1).

**TABLE 2:** ROI level DVR results at the group level.

Parameter	AD mean (SD)	4RT mean (SD)	HC mean (SD)	AD versus 4RT *p*‐value (FDR)	AD > HC *p*‐value (FDR)	4RT > HC *p*‐value (FDR)
Frontal	0.92 (0.11)	0.90 (0.08)	0.86 (0.06)	0.154	**0.010** ^*^	0.145
Occipital	1.03 (0.17)	0.95 (0.08)	0.89 (0.06)	**0**.**000** ^***^	**0.000** ^***^	**0.021** ^*^
Lateral occipital cortex	1.07 (0.22)	0.96 (0.10)	0.88 (0.06)	**0.000** ^***^	**0.000** ^***^	**0.022** ^*^
Parietal	0.97 (0.17)	0.91 (0.09)	0.86 (0.07)	**0.002** ^**^	**0.001** ^**^	0.177
Inferior occipital lobule	1.04 (0.23)	0.93 (0.09)	0.89 (0.07)	**0.000** ^***^	**0.001** ^**^	0.177
Temporal	1.03 (0.13)	0.94 (0.07)	0.91 (0.05)	**0.000** ^***^	**0.000** ^***^	0.139
Inferior temporal gyrus	1.18 (0.18)	1.03 (0.08)	0.98 (0.07)	**0.000** ^***^	**0.000** ^***^	0.111
Middle temporal gyrus	1.10 (0.20)	0.97 (0.09)	0.93 (0.06)	**0.000** ^***^	**0.001** ^**^	0.156
Fusiform temporal gyrus	1.12 (0.14)	1.02 (0.08)	0.97 (0.06)	**0.000** ^***^	**0.000** ^***^	0.139
Posterior cingulate	1.15 (0.22)	1.02 (0.10)	0.99 (0.08)	**0.000** ^***^	**0.000** ^***^	0.296
Anterior cingulate	0.89 (0.10)	0.88 (0.09)	0.86 (0.06)	0.276	**0.035** ^*^	0.335
Insula	0.92 (0.09)	0.90 (0.08)	0.90 (0.05)	0.120	0.157	0.920
Hippocampus	0.94 (0.11)	0.94 (0.10)	0.91 (0.04)	0.839	0.210	0.338
Amygdala	0.93 (0.11)	0.89 (0.11)	0.86 (0.05)	0.075	**0.022** ^*^	0.338
Thalamus	0.98 (0.09)	1.01 (0.09)	0.98 (0.07)	0.159	0.485	0.335
Pallidum	1.08 (0.13)	1.19 (0.13)	1.06 (0.10)	**0.000** ^***^	0.371	**0.011** ^*^
Putamen	1.25 (0.15)	1.33 (0.15)	1.20 (0.11)	**0.007** ^**^	0.238	**0.029** ^*^
Caudate	1.03 (0.09)	1.05 (0.10)	0.99 (0.07)	0.304	**0.047** ^*^	0.099
Accumbens	1.06 (0.10)	1.08 (0.12)	1.00 (0.07)	0.397	**0.035** ^*^	0.067

*Note*: Differences are FDR corrected and controlled for age and sex. Bold values indicate statistically significant results at the *p* < 0.05 level.

Abbreviations: 4RT, 4R‐tauopathy; AD, Alzheimer's disease; FDR, false‐discovery rate; HC, healthy control; ROI, region of interest; DVR, distribution volume ratios.

* *p* < 0.05.

** *p* < 0.01.

*** *p* < 0.001.

**FIGURE 2:**
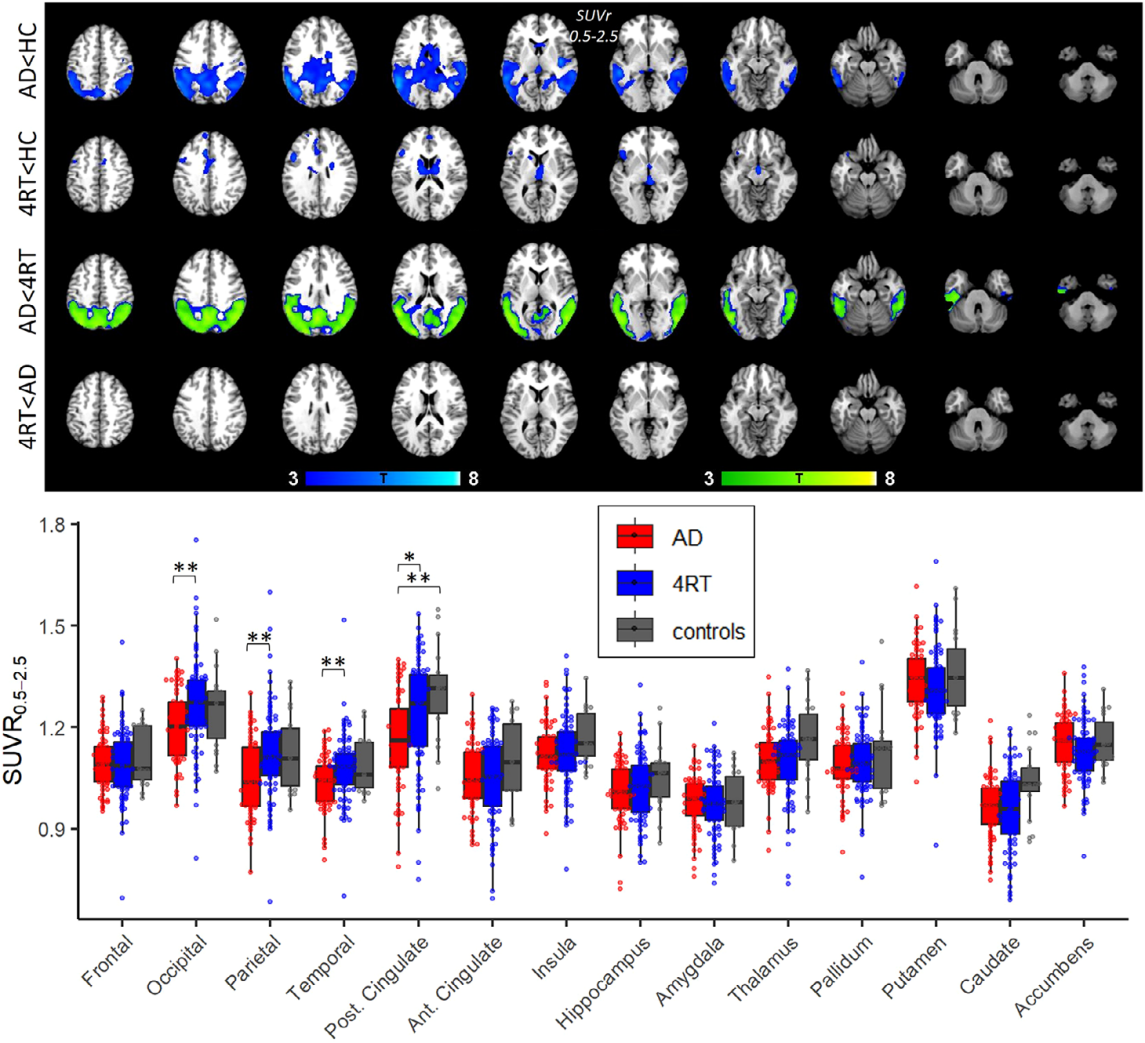
Group differences of perfusion. The top images show voxel‐wise group comparisons of tau binding. Green areas demonstrate FDR‐corrected voxels. Voxel‐wise T‐statistics are shown at *p *< 0.001, *k *> 500, controlled for age and sex, and overlaid on a standard template T1 MRI image. The bottom image shows SUVR_0.5–2.5_ group comparisons in single regions of interests. FDR, false‐discovery rate; MRI, magnetic resonance imaging; SUVr, standardized uptake value ratio.

Next, perfusion characteristics based on early‐phase SUVR_0.5–2.5_ were compared between groups. Voxel‐wise *t*‐tests revealed significantly lower perfusion (hypoperfusion) in AD compared to controls in the parietal, temporal cortex, and caudate (*p *< 0.001, uncorrected, *k *> 500) and compared to 4RT in parietal, occipital, and temporal cortex (*p *< 0.001, FDR corrected, *k *> 500). In 4RT, hypoperfusion was detected in the caudate when compared to controls (*p *< 0.001, uncorrected, *k *> 500) but not when compared to AD (Figure 2).

In the ROI analyses, significant hypoperfusion was found in AD compared to 4RT in posterior cortical regions, and compared to controls in posterior cingulate cortex, inferior parietal lobule, and middle temporal gyrus (Table 3, Figure 2). No significant ROI‐based hypoperfusion differences were found when comparing 4RT to controls after FDR correction. Clinical scales were neither associated with tau‐binding nor perfusion (Supplementary Results).

**TABLE 3:** ROI level SUVR_0.5–2.5_ results at the group level.

Parameter	AD mean (SD)	4RT mean (SD)	HC mean (SD)	AD < 4RT *p*‐value (FDR)	AD < HC *p*‐value (FDR)	4RT < HC *p*‐value (FDR)
Frontal	1.10 (0.08)	1.09 (0.10)	1.12 (0.09)	0.900	0.548	0.749
Occipital	1.21 (0.10)	1.27 (0.14)	1.26 (0.12)	**0.002** ^**^	0.184	0.749
Lateral occipital cortex	1.09 (0.11)	1.16 (0.14)	1.15 (0.11)	**0.001** ^**^	0.092	0.749
Parietal	1.05 (0.11)	1.13 (0.13)	1.13 (0.11)	**0.001** ^**^	0.721	0.806
Inferior occipital lobule	1.01 (0.11)	1.09 (013)	1.11 (0.11)	**0.000** ^***^	**0.007** ^**^	0.999
Temporal	1.04 (0.08)	1.08 (0.10)	1.09 (0.08)	**0.005** ^**^	0.092	0.999
Inferior temporal gyrus	1.04 (0.09)	1.09 (0.11)	1.10 (0.10)	**0.001** ^**^	0.064	0.999
Middle temporal gyrus	0.98 (0.09)	1.03 (0.11)	1.05 (0.08)	**0.002** ^**^	**0.012** ^*^	0.806
Fusiform temporal gyrus	1.16 (0.9)	1.20 (0.11)	1.19 (0.10)	**0.011** ^*^	0.490	0.727
Posterior cingulate	1.16 (0.14)	1.24 (0.16)	1.30 (0.14)	**0.003** ^**^	**0.012** ^*^	0.749
Anterior cingulate	1.05 (0.10)	1.05 (0.13)	1.11 (0.12)	0.849	0.296	0.621
Insula	1.12 (0.09)	1.12 (0.11)	1.18 (0.08)	0.900	0.135	0.617
Hippocampus	1.01 (0.09)	1.03 (0.10)	1.05 (0.10)	0.637	0.307	0.749
Amygdala	0.98 (0.08)	0.97 (0.09)	0.98 (0.09)	0.900	0.807	0.999
Thalamus	1.11 (0.09)	1.10 (0.11)	1.17 (0.11)	0.849	0.096	0.330
Pallidum	1.09 (0.09)	1.10 (0.10)	1.13 (0.13)	0.794	0.223	0.749
Putamen	1.34 (0.10)	1.32 (0.12)	1.36 (0.12)	0.637	0.610	0.617
Caudate	0.97 (0.09)	0.96 (0.12)	1.04 (0.10)	0.811	0.082	0.330
Accumbens	1.15 (0.08)	1.13 (0.09)	1.15 (0.08)	0.391	0.872	0.749

*Note*: Differences are FDR corrected and controlled for age and sex. Bold values indicate statistically significant results at the *p* < 0.05 level.

Abbreviations: 4RT, 4R‐tauopathy; AD, Alzheimer's disease; FDR, false‐discovery rate; HC, healthy control; ROI, region of interest; SUVR, standardized uptake value ratio.

* *p* < 0.05.

** *p* < 0.01.

*** *p* < 0.001.

### 3.2 High p‐tau_181_ and t‐tau levels in patients with AD versus 4RT

We tested CSF biomarker differences across groups and found significantly higher p‐tau_181_ in the AD group (*M *= 82.7, *SD* = 36.7) compared to 4RT (*M *= 50.9, *SD* = 28.3, *p *< 0.001, *d *= 0.97) and to controls (*M *= 52.0, *SD* = 19.0, *p *= 0.029, *d *= 1.05). Significantly higher t‐tau levels were found in AD (*M *= 479, *SD* = 216) compared to 4RT (*M *= 270, *SD* = 198, *p *< 0.001, *d *= 0.91) and to controls (*M *= 242, *SD* = 108, *p = *0.025, *d *= 1.95) (Table 1, Figure S4).

### 3.3 The combination of p‐tau_181_ status and tau binding regions is disease‐specific

Next, we explored how p‐tau_181_ levels predict tau binding and whether there were different association patterns per diagnostic group (AD vs. 4RT). Results of the voxel‐wise multiple regression analyses showed no association between p‐tau_181_ and tau binding in AD or in 4RT (*p *< 0.001, uncorrected, *k *> 500). There was no diagnosis (AD vs. 4RT) by p‐tau_181_ interaction effect on tau binding. In the ROI analyses, we found no positive associations between p‐tau_181_ and DVR in any group at *p *< 0.001 that survived correction for multiple comparisons.

**FIGURE 3:**
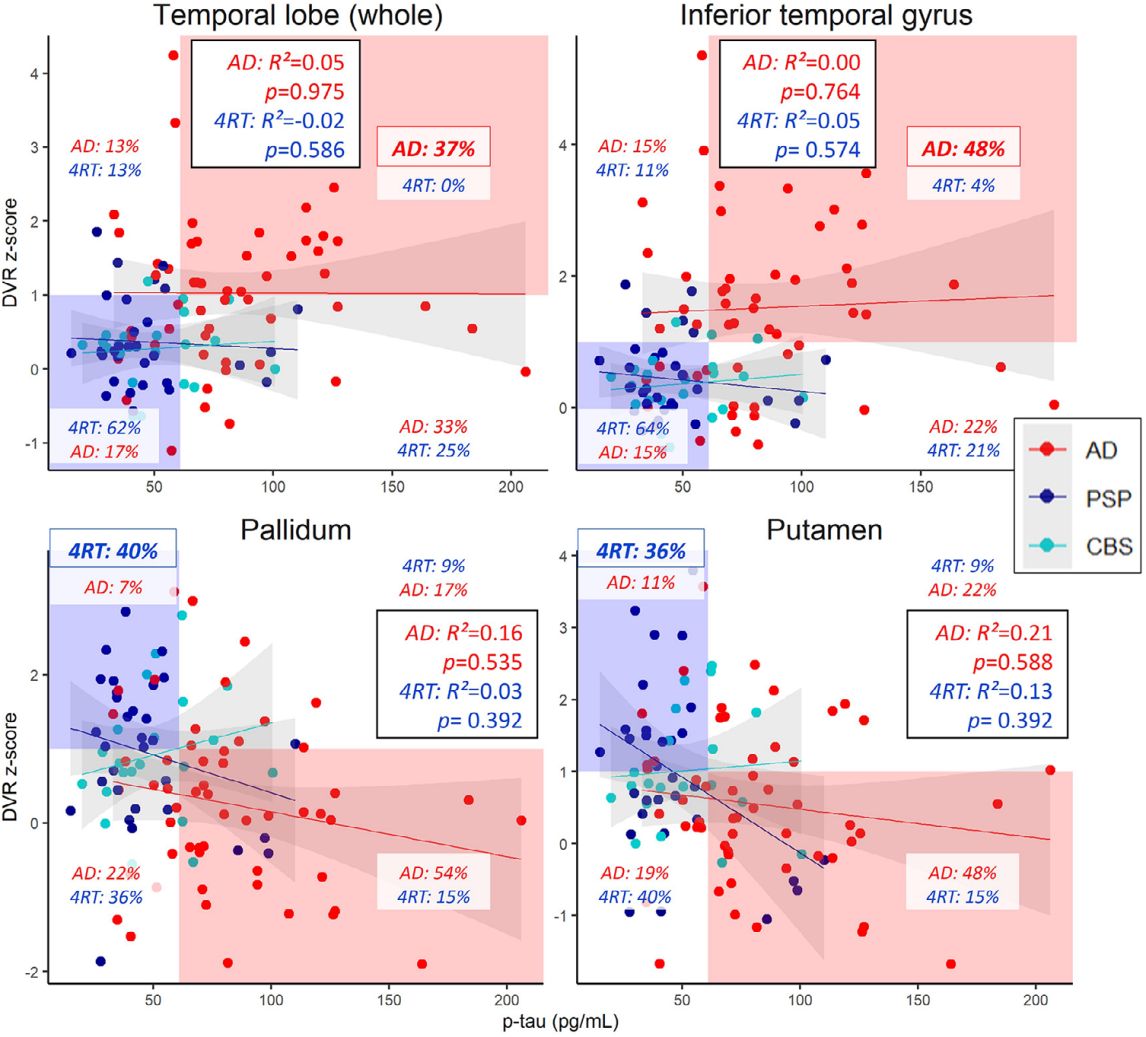
Tau binding distribution in relation to p‐tau_181_. Scatterplots show disease‐specific patterns, highlighting two cortical areas with the strongest PET signal in AD and two subcortical areas with the strongest PET signal in 4RT. Dots and regression lines are shown for PSP and CBS separately. Cutoff values were set to +1 SD (above the mean DVR value of controls) and p‐tau_181_ levels to 61 pg/mL. 4RT, 4R‐tauopathy; AD, Alzheimer's disease; CBS, corticobasal syndrome; DVR, distribution volume ratios; PET, positron emission tomography; PSP, progressive supranuclear palsy.

To further compare the distribution of p‐tau_181_ and tau binding characteristics per group, we calculated the percentage of cases per quadrant of distribution plots in selected ROIs with the strongest disease‐specific PET signal. The whole temporal lobe and the inferior temporal gyrus were chosen as the regions with the strongest tau binding in AD, and the pallidum and putamen were chosen as the strongest tau binding regions in 4RT (Figure 3). Interestingly, most patients with AD showed high p‐tau_181_ in combination with high tau binding in the temporal lobe (*n *= 20, 37%) and in the inferior temporal gyrus (*n *= 26, 48%), compared to only a few or no patients with 4RT showing the same type of combination. Most patients with 4RT showed a combination of normal p‐tau_181_ with high tau binding in the pallidum (*n *= 21, 40%) and putamen (*n *= 19, 36%), while only a few patients with AD showed this type of combination. The proportions were higher when only considering patients diagnosed with PSP (pallidum: *n *= 17, 53%, putamen: *n *= 15, 47%). These different distribution patterns highlight the disease‐specific p‐tau_181_ to tau binding relationships, depending on the brain region.

In a similar way, we explored if t‐tau and hypoperfusion were associated with each other to further test their potential as neuronal injury markers. Results of the voxel‐wise multiple regression analyses revealed no associations between t‐tau and perfusion in any group (*p *< 0.001, uncorrected, *k *> 500). In contrast to our expectation, single ROI analyses indicated significant positive associations in the pallidum (*b *< 0.001, *p *= 0.048, *r^2^ *= 0.20), caudate (*b <* 0.001, *p *= 0.048, *r^2^ *= 0.30), thalamus (*b *< 0.001, *p *= 0.048, *r^2^ *= 0.28), and middle temporal gyrus (*b <* 0.001, *p *= 0.048, *r^2^ *= 0.36) in 4RT, meaning that t‐tau predicted hyper‐perfusion rather than hypoperfusion in these areas. As seen in representative z‐transformed perfusion and t‐tau distribution pattern plots (Figure S5), the positive associations can be explained by generally high perfusion in both patient groups with almost no cases showing perfusion z‐scores lower than −1. There were no disease‐specific differences of distribution.

### 3.4 Tau binding and p‐tau_181_ interplay increases the discriminatory power for AD and 4RT

**FIGURE 4:**
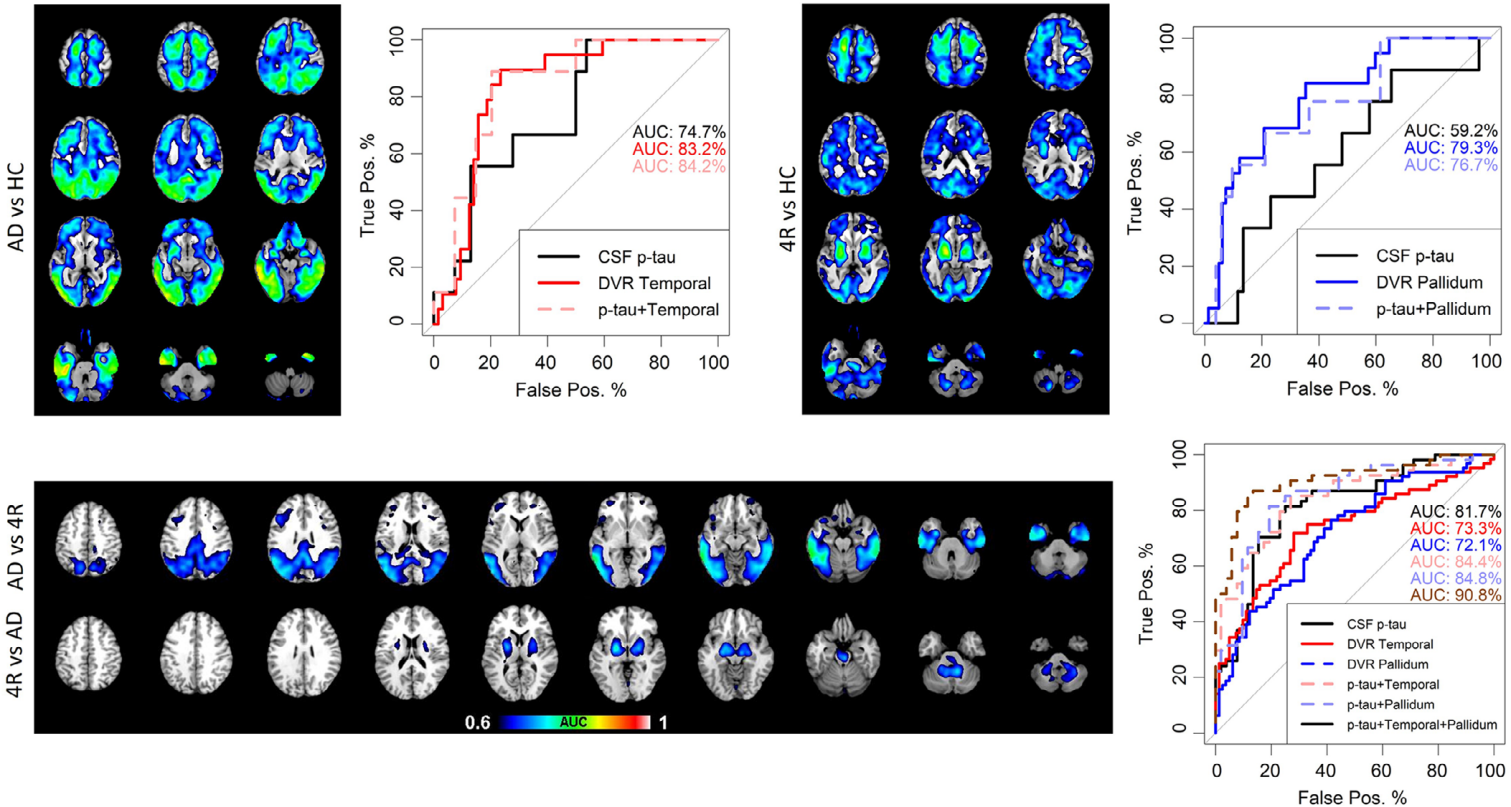
Discriminatory power of tau binding and p‐tau between pairs of groups. Voxel‐wise ROC analysis comparing AD and controls with representative parietal ROI AUC curves (top left); 4RT and controls with representative caudate ROI AUC curves (top right); voxel‐wise discrimination of AD against 4RT and 4RT against AD with parietal and caudate ROI AUC curves, as well as t‐tau and combination of biomarkers (bottom). 4RT, 4R‐tauopathy; AD, Alzheimer's disease; AUC, area under the curve; ROC, receiver operating characteristic; ROI, region of interest.

We explored the diagnostic power of tau markers to discriminate between groups. Voxel‐wise ROC analyses were performed first to detect the most relevant brain regions for the subsequent ROI‐based analyses. Between AD and controls, the voxel‐wise analysis showed that all cortical regions had a high power to discriminate the groups, with the temporal lobe reaching 89%. Between 4RT and controls, the highest discriminatory power was found in the pallidum, which reached 87%. Corresponding ROI‐based AUC curves are shown in Figure 4. AUC curves for other brain regions were either similar, such as the parietal lobe and putamen, or less relevant, such as the frontal region, and were therefore omitted from the manuscript in consideration of conciseness.

Between AD and 4RT, the voxel‐wise analysis showed the highest discriminatory power in temporal regions (AUC 80%) and medium power in parietal, occipital, and frontal regions (AUC 74%). When comparing 4RT against AD, the highest power was observed in the pallidum (AUC 76%) with medium power in the putamen, midbrain, and dentate nucleus (AUC 69%). In the ROI‐based analysis, the AUC of p‐tau_181_ to discriminate the diagnostic groups was 81.7% (95% confidence interval [CI]: 73.5%−89.8%) and when adding to the temporal ROI model, the AUC was 84.4% (95% CI: 77.0%−91.9%) and 84.8% (95% CI: 77.3%−92.3%) when added to the pallidum. When combining p‐tau_181_, temporal, and pallidum tau binding in a model, the discriminatory power was 90.8% (95% CI: 85.0%−96.6%) (Figure 4). Therefore, the combination of p‐tau_181_ and at least one disease‐specific tau binding region exhibited a strong discriminatory power between AD and 4RT, which surpassed the power of a single biomarker alone.

Perfusion characteristics were similarly explored and are described in more detail in the Figure S6. We explored if specific perfusion regions were strong in discriminating the groups, and whether taking t‐tau levels into account increased the diagnostic power. In short, voxel‐wise ROC analyses revealed medium power in the parietal, occipital, and temporal lobe (70%–74%) when discriminating AD from 4RT (AUC 83%) and medium power in the insula (AUC 65%) when discriminating 4RT from AD. The high discriminatory power when combining t‐tau and disease‐specific perfusion markers (up to AUC 80%) was mainly driven by t‐tau (AUC 76.8%).

### 3.5 Adding cortical hypoperfusion to tau biomarkers improves disease discrimination

**FIGURE 5:**
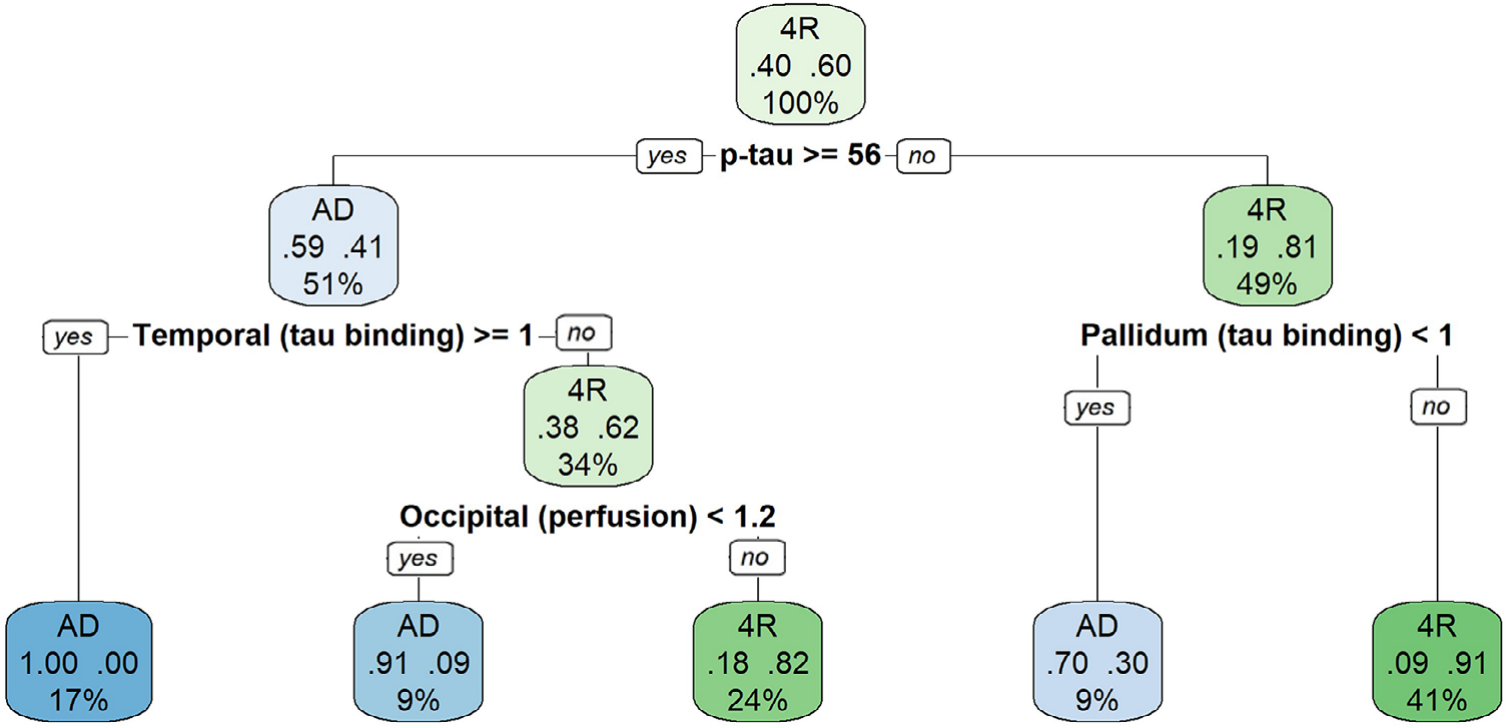
Decision tree analyses to combine tau binding and neuronal injury markers for the detection of AD and 4RT. The analysis included tau tracer binding in the frontal, temporal, parietal, occipital, posterior cingulate, pallidum, and putamen tau tracer binding, as well as p‐tau_181_ as tau indices. Perfusion in the temporal, parietal, occipital, posterior cingulate, caudate, insula, and dorsolateral prefrontal region, and t‐tau were selected as neuronal injury variables. In each square, the left value indicates the probability of being diagnosed with AD, while the right value indicates the probability of being diagnosed with 4RT. 4RT, 4R‐tauopathy; AD, Alzheimer's disease.

As a last step, we investigated if neuronal injury biomarkers would enhance the discrimination of AD and 4RTs, particularly in cases where the tau markers were ambiguous. For the decision tree analysis, we selected the regions with the strongest PET alterations from our findings above. Thus, cortical (frontal, temporal, parietal, occipital, posterior cingulate), pallidum, putamen tau tracer binding, as well as p‐tau_181_, were chosen as tau indices. Perfusion in posterior cortical (temporal, parietal, occipital, posterior cingulate), caudate, insula, and dorsolateral prefrontal region, and t‐tau were selected as neuronal injury variables. The tree selected p‐tau_181_ for the first split. Those cases who showed p‐tau_181_ levels above 56 pg/mL (detected automatically by the model) had a 59% chance of having AD. These cases were further split according to temporal tau binding scores. Those with high tau binding (> 1 DVR scores) had a 100% chance of having AD. If not, they needed to meet the additional criteria of low perfusion in the occipital lobe (< 1.2 SUVR_0.5–2.5_ scores) to have a 91% chance of having AD or would otherwise have 4RT with an 82% chance. On the other side, if those patients who showed p‐tau_181_ levels below 56 pg/mL showed additional high tau binding in the pallidum (> 1 DVR scores), they had a 91% chance of having 4RT, or otherwise a 70% chance of having AD (< 1 DVR scores in pallidum) (Figure 5). The decision tree achieved an accuracy of 82.9%. T‐tau levels and 4RT‐specific perfusion regions were not automatically chosen by the tree model, meaning that their discriminatory performance was negligible. P‐tau_181_ was the primary, most important marker of discrimination.

## 4 DISCUSSION

In this cross‐sectional study, we showed the potential of a diagnostic biomarker‐based algorithm for the discrimination of AD and 4RTs. We demonstrated that a tau biomarker combination of CSF p‐tau_181_ status and ^18^F‐PI‐2620 binding in disease‐specific brain regions is highly useful for separating AD and 4RT. Moreover, ^18^F‐PI‐2620 hypoperfusion served as an additional, supportive neuronal injury biomarker in AD and indicated slight, promising trends in 4RT that merit further investigation. Based on an automatic decision tree splitting, we could show that high p‐tau_181_ levels of >56 pg/mL were the strongest indicator of AD, followed by high temporal tau binding (DVR > 1) and posterior cortical (occipital) hypoperfusion (SUVR_0.5–2.5_ < 1.2). High t‐tau levels (> 445 pg/mL) represented a less strong supportive biomarker of AD. The biomarker‐based diagnostic profile of 4RT was determined by lower p‐tau_181_ levels than AD (< 56 pg/mL) in combination with high basal ganglia (pallidum) tau binding (DVR > 1), but t‐tau and perfusion did not add additional discriminatory value in 4RTs. The specific biomarker‐based algorithm could help determine not only tau pathophysiology in AD, but also in patients with 4RTs, which is useful for future diagnostic workflows and clinical trials.

Similar to previous findings, the tracer ^18^F‐PI‐2620 showed high sensitivity for detecting tau accumulation not only in AD, but also in PSP and CBS.^10^, ^11^, ^18^ Our current study shows typical cortical, mainly temporo‐parietal tau accumulation in AD and dominant globus pallidus accumulation in 4RT, which corresponds to known tau distribution.^1^, ^2^ Elevated ^18^F‐PI‐2620 binding in globus pallidus and putamen of patients with PSP or CBS was found previously.^10^, ^11^, ^18^, ^19^, ^20^ Recent work using ^18^F‐PI‐2620 in 4RTs found that tau pathology patterns in the basal ganglia are associated with brain connectivity^20^ and that lower functional connectivity with cortical regions leads to functional network degradation and disintegration,^19^ reinforcing the idea of trans‐neuronal tau spreading in connected areas.

^18^F‐PI‐2620 in the early scanning phase detected posterior cortical hypoperfusion in AD, and to a lesser extent, hypoperfusion in the caudate, midbrain, and parts of the dorsolateral prefrontal cortex in 4RTs. Early‐phase perfusion SUVr_0.5–2.5_ was previously assessed with ^18^F‐PI‐2620 in our group and revealed cortical and subcortical hypoperfusion in AD and 4RT, which correlated with glucose metabolism of FDG‐PET, R1 of ^18^F‐PI‐2620, or early‐phase Aβ‐PET.^12^, ^13^, ^21^ Although hypoperfusion in the caudate was only slightly observed in 4RT, our findings are consistent with previous studies.^13^ This suggests that early‐phase ^18^F‐PI‐2620 imaging has potential as a supportive marker for neuronal injury in both AD and 4RT. The decision tree analyses confirmed the beneficial role of incorporating cortical hypoperfusion as a biomarker in cases with ambiguous AD profile but suggests that hypoperfusion does not add any diagnostic value in cases where the 4RT diagnosis is uncertain. It is noteworthy that no significant perfusion differences were observed when comparing 4RT against healthy controls and only in the posterior cingulate cortex, inferior parietal lobule, and middle temporal gyrus when comparing AD against controls, which may be attributed to the limited sample size of our control group. The strict correction for multiple comparisons in the current study needs to be acknowledged and should not prevent assessing perfusion in 4RTs since this index already showed promising associations with clinical severity.^13^ Thus, perfusion imaging may be more valuable for 4RT disease monitoring than multimodal discrimination. Preliminary analyses of the current study used the kinetic modeling parameter R1 as a surrogate marker of neuronal damage; however, the results were less robust than early SUVr_0.5–2.5_. The use of the novel tracer for two purposes in a “one‐stop shop” procedure has valuable implications for reducing time, radiation exposure, and patient burden.^13^

Elevated CSF p‐tau_181_ levels in AD in contrast to 4RT are in accordance with previous research, where CSF or plasma p‐tau_217_, p‐tau_181_, or p‐tau_231_ are more specific to AD and elevated in AD compared to primary tauopathies.^22^, ^23^ Our results confirm that p‐tau_181_ levels are not significantly elevated in 4RTs as compared to controls, with most of them showing p‐tau_181_ levels below 61 pg/mL. Positive associations between late‐phase tau‐PET and CSF p‐tau_181_ in AD were previously found in the fusiform^24^ and in cortical areas when using the first‐generation tau‐PET tracer ^18^F‐Flortaucipir.^25^, ^26^ Despite their relationship, tau‐PET and p‐tau should be regarded as two different “T” biomarkers of the original A/T/N system,^7^, ^27^ because fluid markers of soluble p‐tau_181_ species likely reflect a different biological phenomenon than fibrillar tau deposition measured by tau‐PET.^26^, ^28^, ^29^ Recent trials demonstrated a robust association between CSF MTBR‐tau243 and tau‐PET,^30^ underscoring the promising potential of MTBR‐tau243 to detect insoluble tau aggregates and to represent an alternative tau biomarker.

Patients with AD showed elevated CSF t‐tau levels in contrast to the other groups. CSF t‐tau in AD was found to precede positive tau‐PET and to be correlated with CSF p‐tau_181_.^29^, ^31^ It is not a specific marker of AD but rather reflects neuronal injury in various neurological conditions such as in stroke, traumatic brain injury, multiple sclerosis, Huntington's disease, amyotrophic lateral sclerosis, or Creutzfeldt–Jakob disease.^32^, ^33^, ^34^, ^35^, ^36^, ^37^ In the current study, rather than predicting strong hypoperfusion, high t‐tau predicted hyper‐perfusion in the pallidum, caudate, thalamus, and middle temporal gyrus in 4RT. It was previously suggested that each neuronal injury biomarker measures distinct aspects of pathophysiological processes during the disease course.^38^ Here we suggest that CSF t‐tau is not an effective “N” biomarker for differentiating between tauopathies. For future investigations, we suggest neurofilament light chain (NfL) in blood or CSF, which is another widely used fluid biomarker of neurodegeneration, not specific to AD and may be more appropriate for the “N” classification.^39^, ^40^, ^41^, ^42^

A limitation of the study is the lack of autopsy confirmation of the clinically diagnosed 4RT cases, which may have resulted in incorrect diagnoses. In addition, we did not separately analyze different 4RT phenotypes, which would have limited our sample size and therefore statistical power to detect group differences. ^18^F‐PI‐2620 previously found slightly higher tau binding in PSP with Richardson syndrome, compared to patients with other subtypes.^10^ Moreover, although preliminary analyses of the current study excluded potential differences between PSP and CBS, different molecular mechanisms of tau between the two diseases, as well as the absence of tau pathology in some cases of CBS, could lead to different pathological spreading patterns. Yet, basal ganglia pathology represents a typical region for both PSP and CBS.^2^, ^10^, ^11^

The study validated biomarkers on a clinically predefined 4RT cohort. The observed variability in p‐tau_181_ levels and basal ganglia tau binding suggests that the biomarkers might not detect all 4RT cases, some of which may only be identifiable by clinical criteria. Therefore, the proposed biomarker algorithm should primarily serve as a confirmatory tool for cases of 4RT initially diagnosed clinically.

The tracer ^18^F‐PI‐2620 previously showed higher binding affinity to mixed 3R/4RTs,^10^, ^11^, ^43^ due to less favorable kinetically binding strength of ^18^F‐PI‐2620 to tau filaments in 4RTs^44^ and higher clearance rate of the tracer in 4RTs.^43^ The tracer showed less off‐target binding, particularly to monoamine oxidase A and B in brain regions prone to 4R‐tau accumulation (i.e., basal ganglia), a limitation observed with first‐generation tau‐PET tracers such as ^18^F‐Flortaucipir.^45^, ^46^, ^47^, ^48^ ^18^F‐PI‐2620 exhibited high affinity for both 4R‐tau and 3/4R‐tau in autoradiography and immunohistochemistry, along with an improved off‐target binding profile.^9^ Among other second‐generation tau‐PET tracers, only ^18^F‐PM‐PBB3 has shown similarly promising results in binding to 4R‐tau isoforms,^49^, ^50^ though subsequent tests with larger samples and autopsy validations are needed. Our results demonstrate that the ^18^F‐PI‐2620 tracer exhibits high binding characteristics and detects 4R‐tau pathology in the basal ganglia with notable sensitivity and specificity, as was evident from comparing 4RT against both AD and controls.

We cannot be certain that perfusion is an adequate reflection of neuronal injury in our study. Although previous studies have demonstrated that early‐phase tau‐PET perfusion imaging yields results similar to those obtained with FDG‐PET,^21^, ^51^, ^52^ it is important to note that parieto‐occipital hypoperfusion is also observed in Lewy Body Dementia, which limits its specificity as an indicator of AD. FDG‐PET serves as an alternative method for identifying neuronal injury in neurodegenerative diseases, representing a widely used and validated tool for detecting glucose metabolism. Moreover, we recommend that future studies should include a head‐to‐head comparison of early perfusion tau‐PET against MRI to further validate our findings.

Last, we cannot rule out possible confounds due to CSF biomarker fluctuations, as has been reported with diurnal Aβ fluctuations.^53^ We therefore recommend considering possible p‐tau or t‐tau biomarker fluctuations in future clinical trials.

In conclusion, our study was able to show that the combination of disease‐specific tau‐PET binding pattern and CSF p‐tau_181_ status can be used as a reliable diagnostic biomarker‐based algorithm to differentiate between AD and 4RTs. In addition, we showed that early‐phase tau‐PET and, to a lesser extent, CSF t‐tau status could be used as supportive neuronal injury biomarkers for the identification of AD. Novel combinations of biomarkers not only offer hope for more definite detection of non‐AD tauopathies, thereby enhancing current diagnostic approaches with in vivo assessments but also are crucial in using biomarker endpoints to significantly advance future clinical trials.

## CONFLICT OF INTEREST STATEMENT

U.F. reported personal fees from Ipsen and Allergan outside the submitted work. V.V. reported grants from Piramal Imaging during the conduct of the study and personal fees from Shanghai Green Valley outside the submitted work. Dr. Seibyl reported other support from InviCRO and Life Molecular Imaging during the conduct of the study and other support from Roche and Biogen outside the submitted work. O.S. received research support from LMI. H.B. received reader honoraria from LMI. F.S. received honoraria for advisory boards of Amylyx, Alnylam, and Alexion and author fees from W. Kohlhammer GmbH medical publishers. A.Z. received speaker fees and research support from Dr. Willmar Schwabe GmbH. G.U.H. has ongoing research collaborations with Roche, UCB, Abbvie; serves as a consultant for Abbvie, Alzprotect, Amylyx, Aprineua, Asceneuron, Bayer, Bial, Biogen, Biohaven, Epidarex, Ferrer, Kyowa Kirin, Lundbeck, Novartis, Retrotope, Roche, Sanofi, Servier, Takeda, Teva, UCB; received honoraria for scientific presentations from Abbvie, Bayer, Bial, Biogen, Bristol Myers Squibb, Kyowa Kirin, Pfizer, Roche, Teva, UCB, Zambon; holds a patent on Treatment of Synucleinopathies (US 10,918,628 B2, Date of Patent: Feb. 16, 2021; EP 17 787 904.6‐1109 / 3 525 788); received publication royalties from Academic Press, Kohlhammer, and Thieme. J.L. reports speaker fees from Bayer Vital, Biogen, and Roche; consulting fees from Axon Neuroscience and Biogen; author fees from Thieme medical publishers and W. Kohlhammer GmbH medical publishers; non‐financial support from Abbvie; and compensation for duty as part‐time CMO from MODAG, all outside the submitted work. M.B. received speaker honoraria from GE healthcare, Roche, and LMI and is an advisor of LMI. N.F. received speaker honoraria from Eisai and LMI and consulting honoraria by MSD. All other authors declare that they have no conflicts of interest. Author disclosures are available in the supporting information.

## CONSENT STATEMENT

Informed consent was obtained from all patients. The study was conducted in accordance with the principles of the Declaration of Helsinki. Approval for scientific data analysis was obtained from the local ethics committee (application numbers 17‐569, 19‐022).

## Supporting information

Supporting Information

Supporting Information
