# Supplementary material for: Combining cerebrospinal fluid and PI‐2620 tau‐PET for biomarker‐based stratification of Alzheimer's disease and 4R‐tauopathies
Source: Alzheimers Dement. 2024 Sep 12;20(10):6896–909. doi: 10.1002/alz.14185 (PMC11485081; doi:10.1002/alz.14185)
Supplement: Supplementary file 1 — Supporting Information [file ALZ-20-6896-s002.docx]

**Supplementary Content**

## **Supplementary Results:**

## Clinical scales are not associated with tau-binding or perfusion

When testing whether PET measures of tau and perfusion predicted clinical severity, no associations were found between DVR or early-phase SUVr and either MoCA, PSPRS, UPDRS, or SEADL in any group after correction for multiple comparisons. In the AD group, MoCA scores were significantly lower (*M*=17.6, *SD*=6.4) compared to 4RT (*M*=22.1, *SD*=4.7, *p*<0.001, *d*=0.81) and to controls (*M*=27.8, *SD*=2.1, *p*<0.001, *d*=2.15).

## **Supplementary Figures:**

**Supplementary Figure 1**


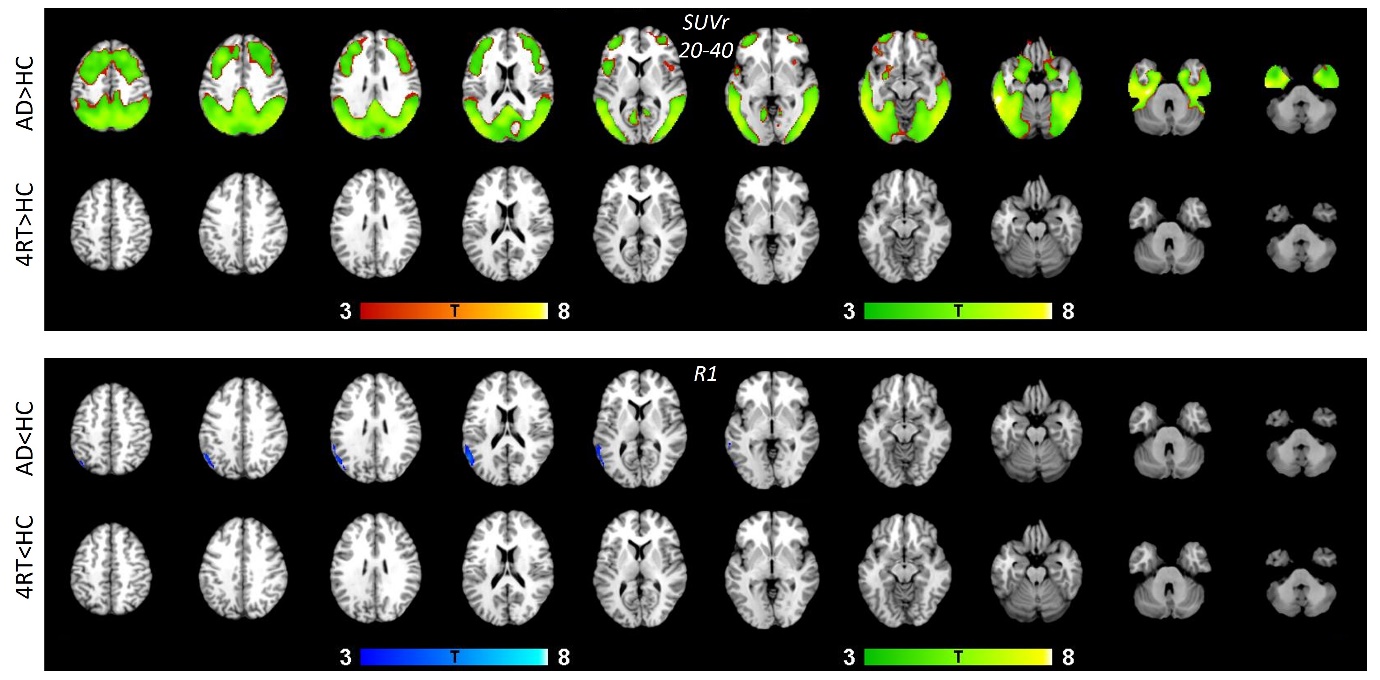


**Supplementary Figure 1. Group differences of tau binding and blood flow.** Top images show increased cortical tau uptake (SUVr 20-40 min) in AD compared to controls, while no difference was found when comparing 4RT to controls. The bottom images show decreased blood flow (R1) in parts of the parietal lobe in AD compared to controls, but no significant difference when comparing 4RT to controls. Green areas demonstrate FDR-corrected voxels. Voxel-wise T-statistics are shown at *p*<0.001, *k*>500, controlled for age and sex, and overlaid on a standard template T1 MRI image.

**Supplementary Figure 2**


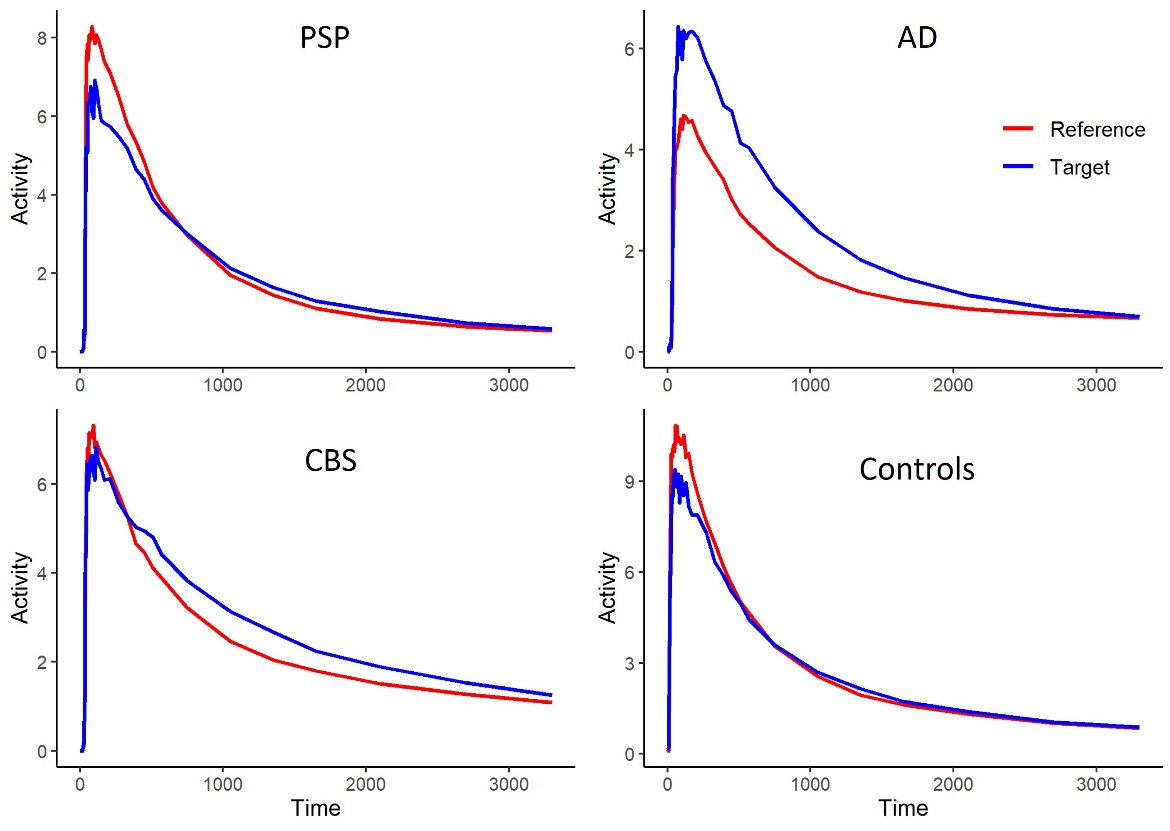


**Supplementary Figure 2. Time-Activity Curves for four randomly selected individuals of each group.** The blue curves represent the target/overall tau-PET signal over time, with the individual time-activity curve of the reference region.

**Supplementary Figure 3**


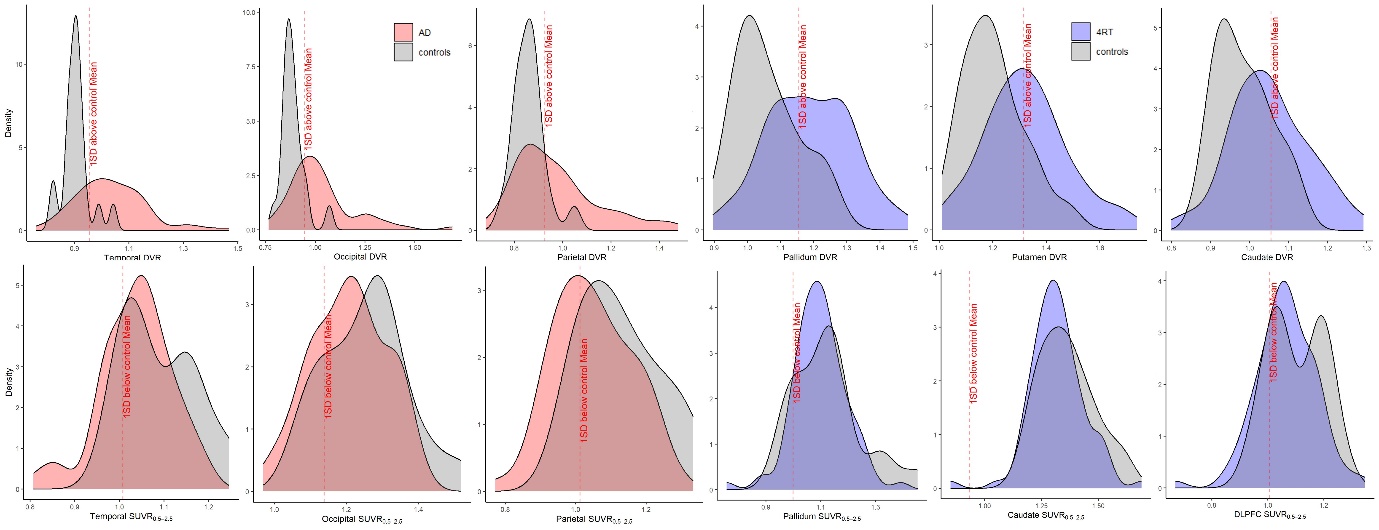


**Supplementary Figure 3. Density plots for SD threshold definition.** The top row shows density plots for DVR, and the bottom row shows early-phase SUVr (0.5-2.5 min). Red areas represent the distribution in the AD group, and blue areas represent the 4RT group. The dotted red line corresponds to the +1 SD cut-off above the control mean. The DVR, particularly in AD, the +1 SD cut-off effectively shows minimal false positives, as indicated by the small overlapping area on the right side of the red line. In contrast, the SUVr density plots show significant overlap, indicating that -1 SD is less effective as an ideal cut-off value.

**Supplementary Figure 4**


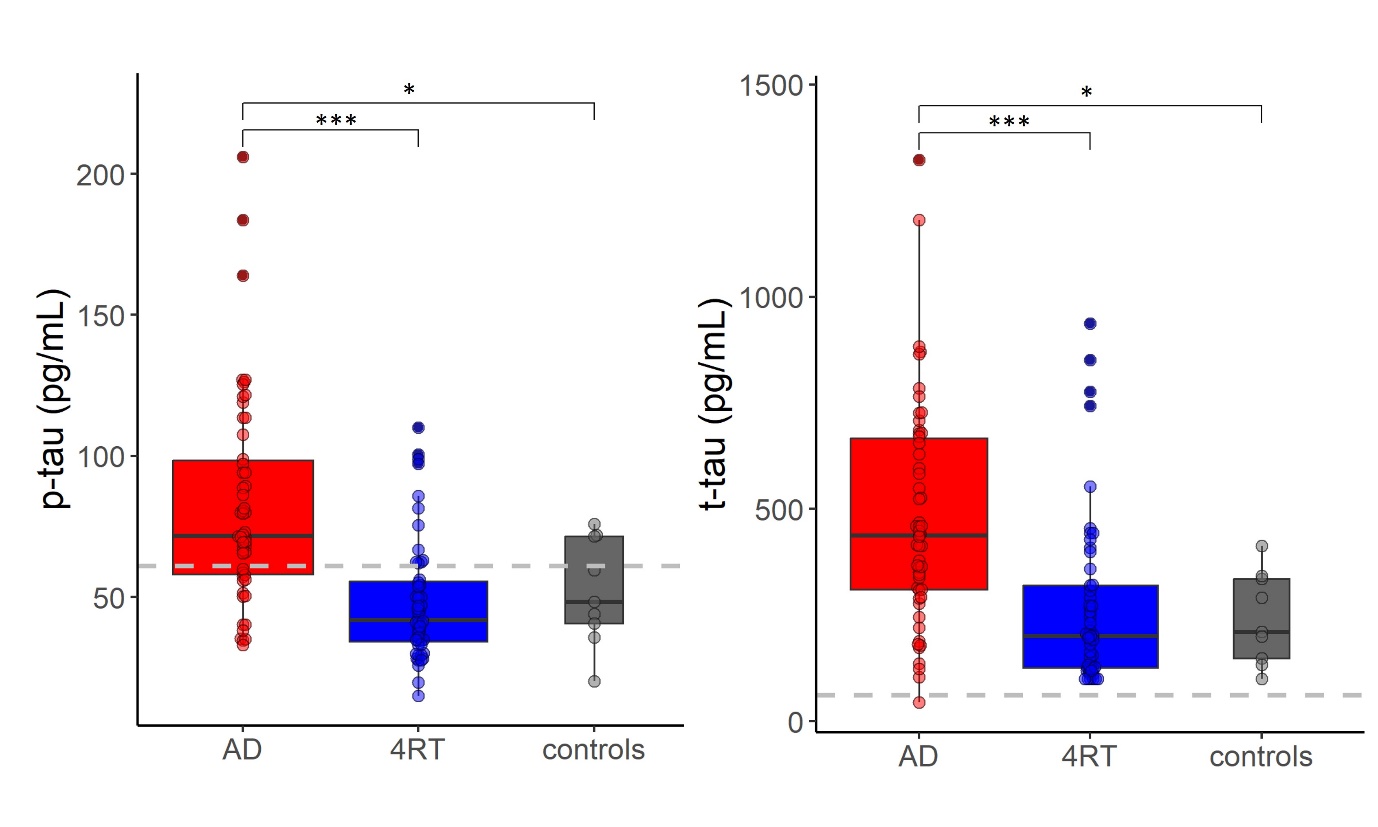


**Supplementary Figure 4. Group differences of CSF.** Asterisks indicate post-hoc p-values based on Tukey tests applied to ANCOVAs (with age and sex as covariates), showing higher p-tau_181_ and t-tau levels in AD compared to 4RT and to controls.

**Supplementary Figure 5**


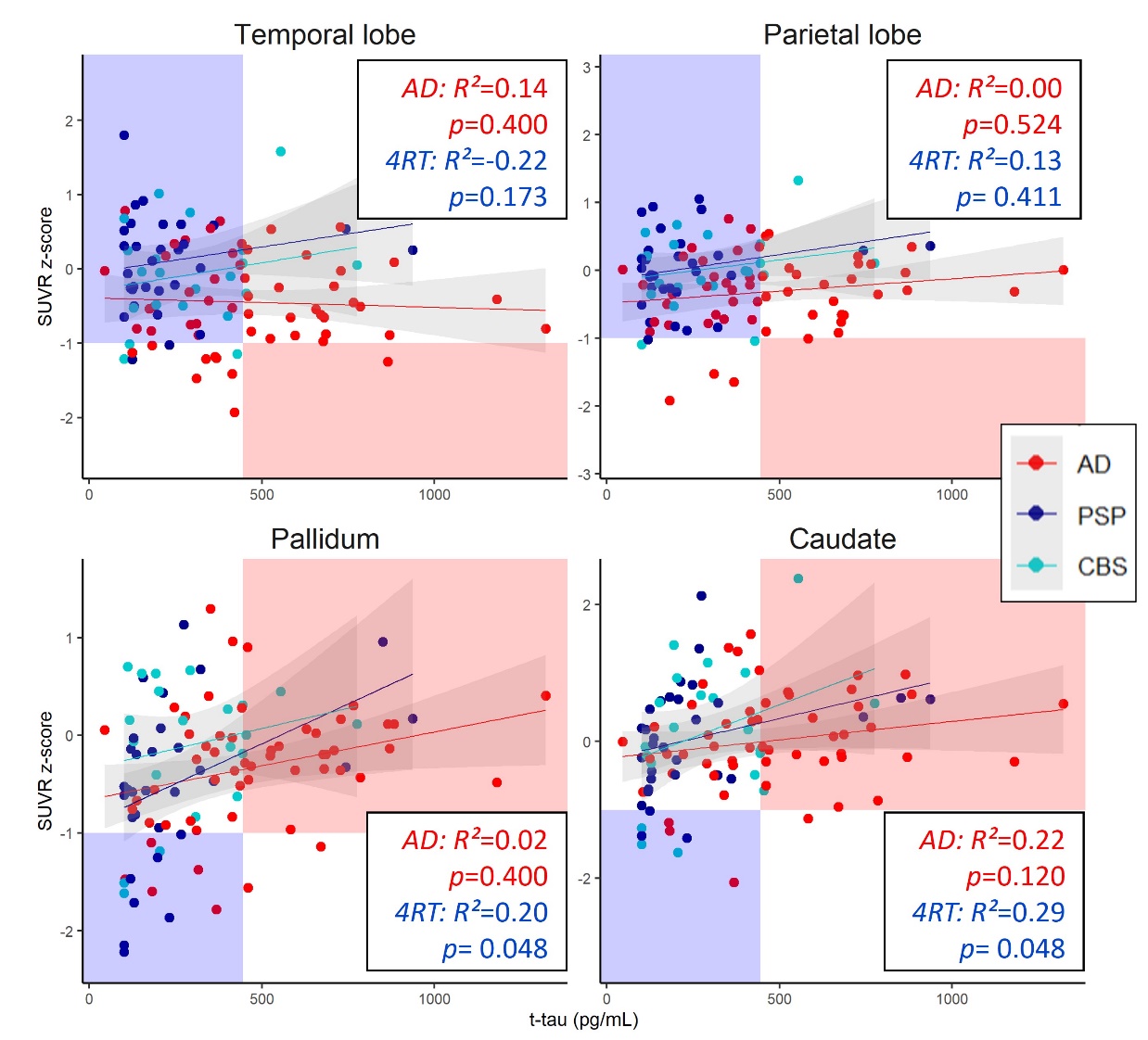


**Supplementary Figure 5. Perfusion distribution in relation to t-tau.** Scatterplots show no disease-specific combination or association between t-tau and early-phase tau-PET SUVr in any ROI. Cut-off values were set to -1 SD (below the mean SUVr value of controls) and t-tau levels to 445 pg/mL. Only a few AD patients showed low perfusion (SUVR_0.5-2.5_­­­ z-scores) combined with high t-tau in the temporal lobe (*n=*1, 2%). Few 4RT patients showed low perfusion combined with low t-tau in the pallidum (*n*=6, 11%). The z-transformed values indicate that both patient groups generally exhibit normal to higher (i.e. hyper-perfusion) than average perfusion.

**Supplementary Figure 6**


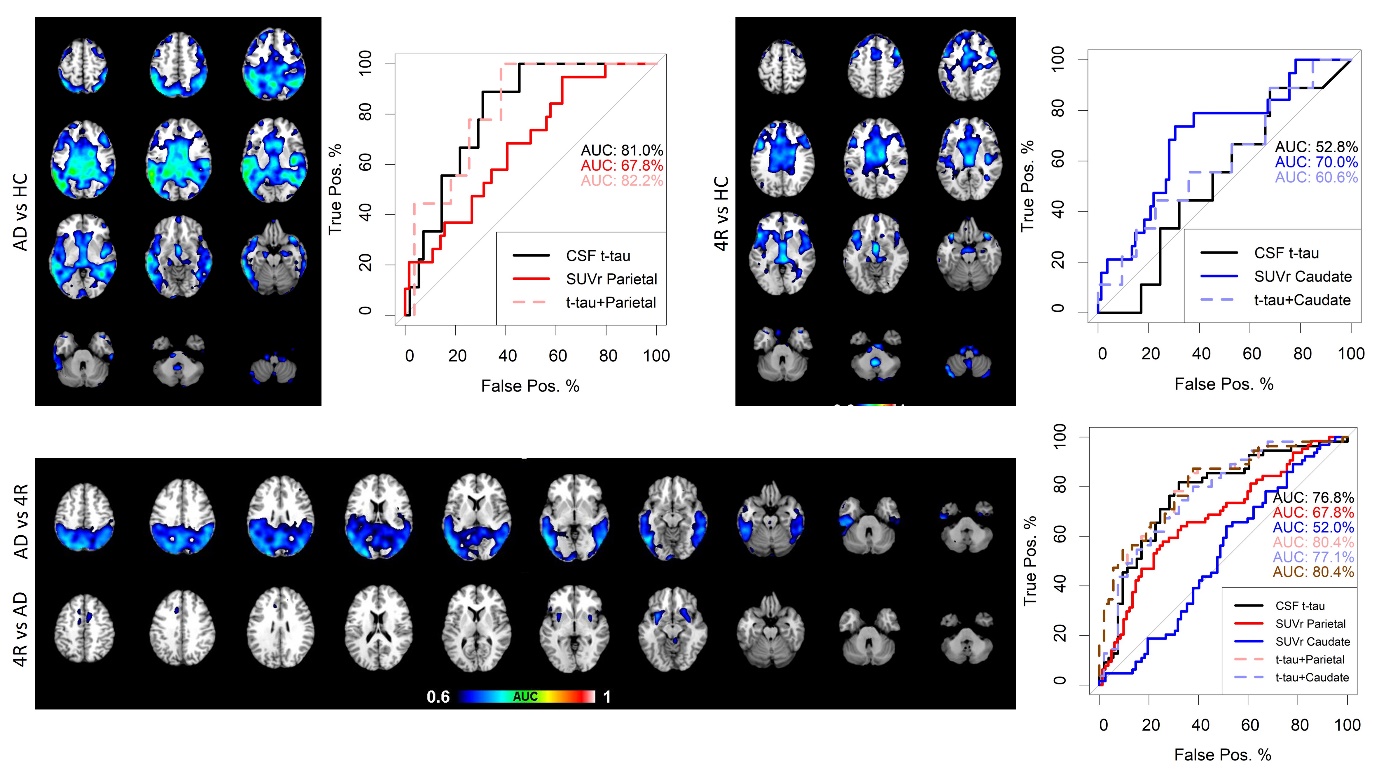


**Supplementary Figure 6. Discriminatory power of perfusion and t-tau between groups.** Between AD and controls (top left): Voxel-wise ROC analysis showed high discriminatory power of hypoperfusion in the parietal lobe (AUC up to 83%) and medium power in the posterior cingulate cortex and caudate (AUC 75%)**.** The representative parietal ROI ROC analysis is shown to the right. Between 4RT and controls (top right): Voxel-wise ROC analysis showed the highest discriminatory power of hypoperfusion in the midbrain, caudate, and parts of the dorsolateral prefrontal cortex (AUC 75%)**.** The representative caudate ROI ROC analysis is shown to the right. Discrimination of AD against 4RT (bottom): Voxel-wise ROC analysis showed high power in the parietal, occipital, and temporal lobe (AUC 70-74%). Discrimination of 4RT against AD: The highest discriminatory power was found in the insula (AUC 65%). The AUC of t-tau was 76.8% (95% CI: 67.7%-86.0%). Adding t-tau to the parietal model increased the AUC to 80.4% (95% CI: 72.2%-88.6%) and to 77.1% (95% CI: 68.1%-86.0%) in the caudate model (insula: 76.8% (95% CI: 67.9%-85.9%)). Combining t-tau, parietal, and caudate markers resulted in an AUC of 80.4% (95% CI: 72.3%-88.6%), driven by the high discriminatory power of t-tau.
